# Administrative interventions in antimicrobial stewardship: a global scoping review of all WHO regions

**DOI:** 10.3389/fphar.2026.1726284

**Published:** 2026-05-08

**Authors:** Ali Uzair, Anees Ur Rehman, Zikria Saleem, Masaad Saeed Almutairi

**Affiliations:** 1 Department of Pharmacy Practice, Faculty of Pharmacy, Bahauddin Zakariya University Multan, Multan, Pakistan; 2 Department of Pharmacy Practice, College of Pharmacy, Qassim University, Qassim, Saudi Arabia

**Keywords:** administrative stewardship, antibiotic policy, antibiotic resistance, antibiotic stewardship, antibiotic use, antibiotics, antimicrobial resistance, framework

## Abstract

Although global antimicrobial resistance strategies increasingly emphasize multisectoral and systems-level responses, the role of administrative and governance-driven interventions within antimicrobial stewardship (AMS) remains relatively underexplored in the literature. Successful antimicrobial stewardship programs (ASPs) can only be achieved through multi-stakeholders’ efforts, administration, and leadership at a multifaceted health system level, involving interventions that engage healthcare providers and patients and are supported by governance commitment and backed by financial support. The aim of this scoping review was to identify and map global evidence on administrative AMS interventions that shape ASPs. A systematic search of major databases (Google Scholar and PubMed) was conducted for studies published between January 2005 and June 2025. A total of 76 studies across all the World Health Organization (WHO) regions met the inclusion criteria and were included in the review. The included studies comprised observational studies, quasi-experimental studies, randomized control trials, and mixed-method studies. Data were extracted, mapped, and structured into six thematic categories pertaining to administrative AMS interventions: 1. health-system administrative governance, 2. community-centered administrative strategies, 3. prescription rationalization and dispensing regulation, 4. institutional accountability and premiums, 5. technology-driven education and capacity building, and 6. infection prevention measures. The findings of this study highlight the crucial role of governance-driven administrative interventions in strengthening antimicrobial stewardship and provide policy-relevant insights for augmenting administrative stewardship strategies globally.

## 1 Introduction

Antimicrobial resistance (AMR) has emerged as one of the most serious public health challenges of our time, affecting people, communities, and healthcare systems worldwide (WHO, 2021). Higher morbidity, mortality, extended hospital stays, and increased medical expenses are closely linked to increasing antibiotic resistance (Cosgrove, 2006). AMR is an established global public health crisis, responsible for more than 1.2 million deaths annually (Murray et al., 2022), and the World Health Organization (WHO) has proclaimed AMR as one of the top 10 global health threats facing humanity today (Organization, 2021). In 2021, an estimated 4.71 million deaths were associated with bacterial AMR, including 1.14 million deaths directly attributable to bacterial AMR (Naghavi et al., 2024). If current trends continue, antimicrobial resistance could directly cause 1.9 million deaths and contribute to a total of 8.2 million deaths globally by 2050 (Naghavi et al., 2024) and an annual economic burden of 100 trillion dollars (O’neill, 2014). According to WHO, AMR propagation is unequalled, impacting high-income countries (HICs), upper–middle-income countries (UMICs), low–middle-income countries (LMICs), and low-income countries (LICs) (Akpan et al., 2020). Several biological and social factors influence AMR; however, it is principally driven by the overuse and misuse of antibiotics by patients and injudicious prescribing by healthcare providers (G et al., 2022). The global antibiotic consumption rate has increased by 46% from 9.8 defined daily doses (DDDs) per 1,000 population per day in 2000 to 14.3 DDDs per 1,000 per day in 2018 (Browne et al., 2021).

Nevertheless, this global increase in antibiotic consumption is driven by multiple factors, including extensive use (Aljarallah, 2017), inappropriate prescribing practices, over-the-counter access, and broader health system inefficiencies (Garg et al., 2014; Li et al., 2012).

To combat AMR, a number of countries, including China, have focused on preserving the effectiveness of last-line antibiotics by forbidding their use in animal feed (Heffernan, 2022). However, such initiatives alone do not suffice to curtail the rapid spread of AMR (Ogunlana et al., 2023). To achieve a comprehensive success against AMR, impactful health system level interventions are required (Moyo et al., 2023). To address this, the World Health Assembly’s Global Action Plan on Antimicrobial Resistance (2015) has identified judicious use of antibiotics as one of its five main goals, requiring immediate intervention (Organization, 2015). WHO has highlighted six essential health system level interventions to address AMR, namely, impactful antimicrobial leadership, capacity building through continuous training and education for providers and patients, development and enforcement of antimicrobial guidelines and protocols, regular feedback on antibiotic use, transparent reporting systems, and well-defined accountability and responsibility frameworks (Oxford Academic, 2015).

Successful implementation of antimicrobial stewardship (AMS) programs (ASPs) requires the efforts of multiple stakeholders and multifaceted interventions involving healthcare providers and patients, supported by effective leadership, governance commitment, and financial backing. Most of the existing evidence on AMS interventions focuses on clinical tools, such as audit and feedback (Baur et al., 2017), guideline adherence (Davey et al., 2013), education and awareness (Charani et al., 2011; Cox et al., 2017), prescriber’s behavior (Schuts et al., 2016; Pulcini et al., 2017), surveillance, campaigns, and access to antimicrobials (Huttner et al., 2010; Basnyat et al., 2015; Krockow et al., 2022; Howard et al., 2015), yet these studies generally analyze interventions from a clinical perspective, targeting individual healthcare providers. However, the role of leadership and administrative stewardship, with the health system as the unit of analysis, in enabling or constraining these clinical efforts has been comparatively underexplored.

**FIGURE 1:**
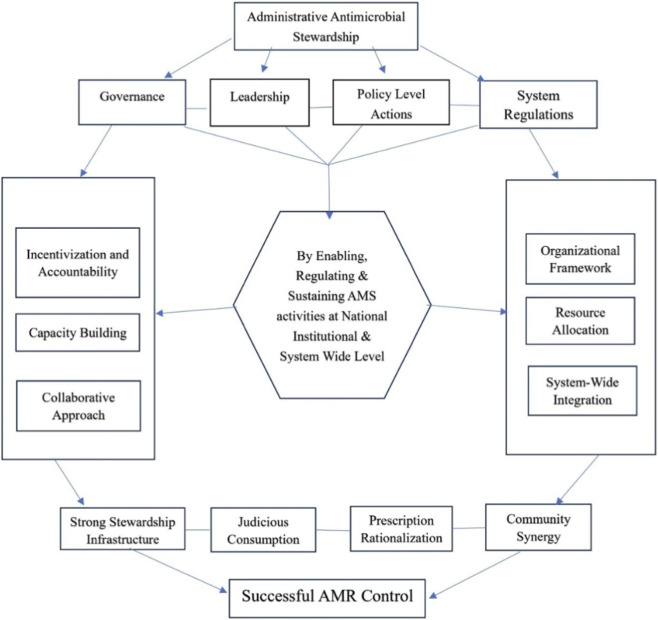
Conceptual model to define administrative antimicrobial stewardship.

The importance of our study lies in the fact that it shifts the paradigm and scale of AMS beyond the clinical level to the administrative and leadership domains. Through the systematic structuring of global evidence, this study indicates how administration- and leadership-enabled health system governance, institutional accountability, and community-centered strategies collectively shape stewardship outcomes. To our knowledge, administrative AMS is not categorically defined in existing evidence bases. This review addresses this gap by mapping evidence from multiple WHO regions, categorizing administrative interventions into thematic areas, and structuring a conceptual model (Figure 1) to advance its definition within the broader discourse on antimicrobial resistance governance.

### 1.1 Aim of this scoping review

The primary aim of this scoping review was to map and synthesize global evidence on administrative interventions that impact ASPs. The focus is on understanding how leadership, policy, institutional, and governance decisions of health systems shape stewardship activities and contribute to more judicious antimicrobial consumption. Our intention is to provide policymakers with actionable insights to develop system-wide policy reforms aimed at establishing and strengthening administrative AMS to curb AMR.

## 2 Materials and methods

Our scoping review was carried out following the Preferred Reporting Items for Systematic Reviews and Meta-Analysis extension for scoping reviews (PRISMA-ScR) guidelines (Tricco et al., 2018).

### 2.1 Selection criteria

The inclusion criteria for studies were as follows: 1. publications in English; 2. involvement of healthcare settings [hospitals, general practitioner (GP) clinics, and nursing care homes], communities, and policymakers, where antimicrobial stewardship interventions are implemented; 3. studies examining administrative AMS- or AMR-related interventions implemented in any country or health system worldwide; 4. empirical peer-reviewed full-text studies; 5. studies examining administrative AMR interventions and their impact; and 6. publications from 2005 onwards.

The exclusion criteria for studies were as follows: 1. publications in languages other than English; 2. studies focused on individual prescribers, patients, or other clinical staff without administrative involvement; 3. publications not linked to an administrative body, healthcare facility, or a formal health system; 4. opinions, commentaries, editorials, letters to the editor, narrative reviews, book chapters, unpublished research, or articles where the full text is not accessible; 5. antimicrobial stewardship programs in animal, agricultural, and environmental settings; 6. studies not examining administrative AMS interventions and their impact; 7. publications before 2005; and 8. duplicate studies.

### 2.2 Search strategy

**FIGURE 2:**
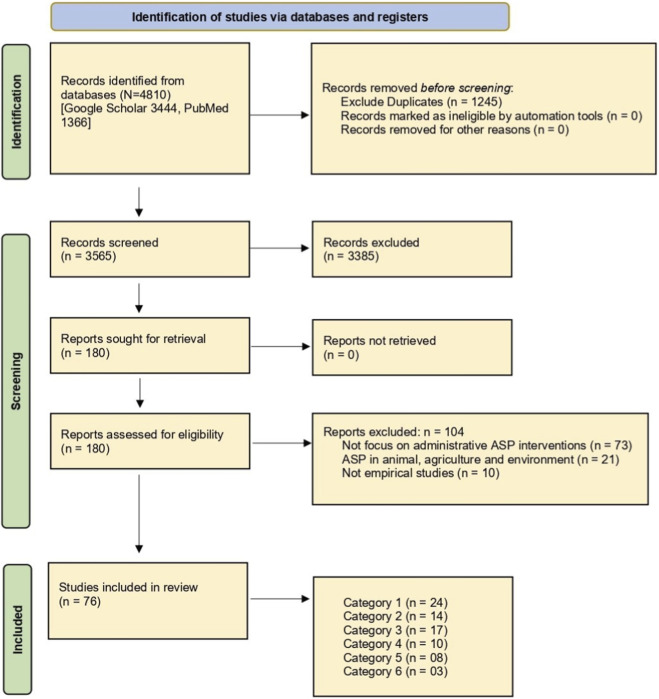
PRISMA‐ScR flow diagram, illustrating the study selection process, including identification, title and abstract screening, full-text assessment, and final inclusion of studies. PRISMA-ScR, Preferred Reporting Items for Systematic reviews and Meta‐Analyses extension for Scoping Reviews; ASP interventions, antimicrobial stewardship program interventions.

Our search strategy was structured using a mix of keywords and Medical Subject Heading (MeSH) terms to identify relevant articles published between January 2005 and June 2025. Two databases (Google Scholar and PubMed) were searched. The search was performed on 30 June 2025, limited to English-language publications, and no study design filters were applied to ensure comprehensive retrieval of relevant evidence. We used a combination of first- and second-string terms in our search mechanism. Keywords such as “administrative decisions,” “administrative intervention,” “hospital administration,” “healthcare leadership,” “institutional strategies,” “healthcare governance,” “executive decisions,” “health managers’ decision,” and “organizational support” were included in the first string. The second string comprised relevant keywords for antimicrobial stewardship, such as “antimicrobial,” “antibiotic stewardship,” “antimicrobial resistance,” “AMS program,” and “antimicrobial consumption.” To refine our search outcomes, we combined these search terms using Boolean operators “OR” and “AND.” The following search commands were used to find articles from the databases: (Administrative decisions OR interventions OR administrative intervention OR hospital administration OR healthcare leadership OR institutional strategies OR healthcare governance OR executive decisions OR Health managers’ decision OR organizational support) AND (Antimicrobial/antibiotic stewardship OR antimicrobial resistance OR AMS program OR antimicrobial consumption/use) AND (Global health OR worldwide OR hospital OR community OR outreach OR community centers OR health system* OR “communication” education or training OR “capacity building” OR orientation OR campaign OR surveillance OR sanitation OR hygiene OR “infection prevention control” OR IPC OR “one health” AND “High-income countries OR low- and middle-income countries OR LMIC”). All retrieved records underwent title and abstract screening prior to full-text eligibility assessment, as illustrated in Figure 2. The PRISMA flow diagram (Figure 2) illustrates the study selection process, including identification, title and abstract screening, full-text assessment, and final inclusion of studies. Records were identified through PubMed and Google Scholar, followed by duplicate removal, screening, eligibility assessment, and inclusion based on predefined criteria. Records identified from PubMed and Google Scholar were screened at the title and abstract level, followed by the full-text review based on predefined inclusion and exclusion criteria.

## 3 Results

We initially retrieved 4,810 relevant articles through comprehensive and systematic database searches in Google Scholar (3,444) and PubMed (1,366). After removing 1,245 duplicates, 3,565 records remained for screening. Title and abstract screening resulted in the identification of 180 relevant articles, of which 76 met the inclusion criteria. One-hundred and four articles were excluded based on the exclusion criteria (Figure 2).

**FIGURE 3:**
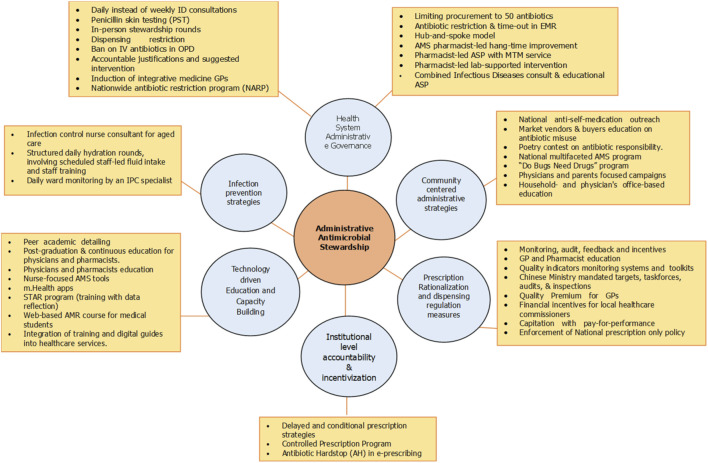
Summarization of some administrative AMS interventions identified in the included studies. The intervention terms reflect descriptions used in the source literature and represent governance, regulatory, and organizational strategies. EMRs, electronic medical records; ASP, antimicrobial stewardship program; MTM, medication therapy management; ID, infectious disease; IV, intravenous; OPD, outpatient department; GPs, general practitioners; AMS, antimicrobial stewardship; STAR, stemming the tide of antibiotic resistance; AMR, antimicrobial resistance; IPC, infection prevention and control.

Administrative decisions to curb AMR include a diverse range of interventions that shape stewardship activities and contribute to more judicious antimicrobial consumption in communities and hospitals. Data extraction was conducted using a standardized data charting form developed for this review. Information extracted included the country name, WHO region, study location, study design, study population, identified administrative intervention, and the impact of the intervention relevant to antimicrobial stewardship. The screening and data extraction process was conducted by the author and verified through repeated cross-checking to ensure accuracy and consistency. After a comprehensive review of the selected articles, administrative AMS interventions in health systems were synthesized and categorized into six categories (Figure 3): 1: health-system administrative governance, 2: community-centered administrative strategies, 3: prescription rationalization and dispensing regulation, 4: institutional accountability and premiums, 5: technology-driven education and capacity building, and 6: infection prevention measures. Categories were based on their primary locus of control and mechanism of action within the health system, with particular emphasis on administrative enablement and system-level implementation pathways. Specifically, interventions were grouped according to whether they operated through governance and policy-level regulation, institutional accountability structures, prescribing behavior modification, technology-enabled system support, community engagement, or regulatory and dispensing controls. Where interventions spanned multiple domains, classification was based on the dominant implementation pathway and intended system-level impact. The conceptual definitions of each category are presented in Table 1. Several interventions identified in this review may be clinical or educational in their direct function. However, within the context of this review, these interventions were classified as administrative AMS strategies when their implementation, scaling, or sustainability was enabled by policy decisions, institutional mandates, or governance-level actions. Thus, the categorization reflects an administrative lens, focusing on the system-level mechanisms that drive and support these interventions, rather than their intrinsic operational nature.

**TABLE 1:** Detailed numbers of included studies.

Characteristic	Category	No. of studies
Country-wise	United Kingdom	10
	China	5
	United States	5
	Netherlands	4
	Canada	4
	Brazil	4
WHO’s regions-wise	EURO (Europe region)	33
	AMRO (Americas region)	15
	WPRO (Western Pacific region)	11
	SEARO (South–East Asia region)	9
	AFRO (Africa region)	5
	EMRO (Eastern Mediterranean region)	3
Study design-wise	Quantitative	56
	Qualitative	11
	Mixed-method	9
Country’s income level-wise	High-income countries (HICs)	45
	Upper–middle-income countries (UMICs)	16
	Lower-income countries (LICs)	8
	Lower–middle-income countries (LMICs)	7
Thematic category-wise	Health-system administrative governance interventions	24
	Prescription rationalization and dispensing regulation measures	17
	Community-centered administrative strategies	14
	Institutional accountability and premiums	10
	Technology-driven education and capacity building	8
	Infection prevention measures	3

**FIGURE 4:**
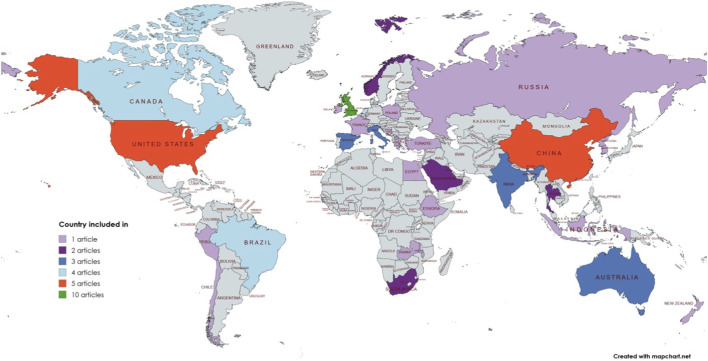
Geographical distribution of contributing countries. Only nations with included studies are colored and labeled; all other landmasses are omitted from the visualization to focus on the evidence base.

**FIGURE 5:**
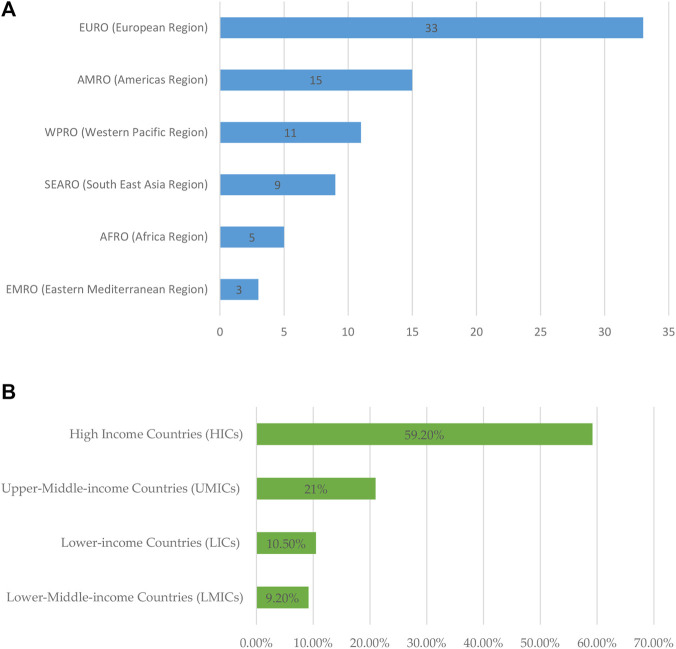
**(A)** Bar chart showing the number of included studies based on WHO regions. **(B)** Bar chart showing the percentage distribution of included studies based on the classification of countries by income.

The studies included in the review were conducted in 37 countries, with the United Kingdom (13.1%), China and the United States (6.5%), and the Netherlands, Canada, and Brazil (5.2%) being the most represented (Figure 4). WHO’s EURO (Europe region) had the highest number of articles (43.4%), followed by AMRO (Americas region) (19.7%), WPRO (Western Pacific region) (14.4%), SEARO (South–East Asia region) (11.8%), AFRO (Africa region) (6.5%), and EMRO (Eastern Mediterranean region) (3.9%). In terms of study design, most studies were quantitative (73.6%), followed by qualitative (14.4%) and mixed-method (11.8%) Figure 5A.

Most of the studies were reported from HICs (59.2%), followed by UMICs (21%), LICs (10.5%), and LMICs (9.2%). The majority of studies (31.5%) reported the administrative stewardship related to health-system administrative governance interventions, followed by prescription rationalization and dispensing regulation measures (22.3%), community-centered administrative strategies (18.4%), institutional accountability and premiums (13.1%), technology-driven education and capacity building (10.5%), and infection prevention measures (3.9%) (Figure 5B). Detailed numbers for each category are provided in Table 2.

**TABLE 2:** Conceptual definition categories.

Administrative AMS category	Conceptual definition
Health-system administrative governance	Interventions operating at the policy or system level that establish regulatory frameworks, national strategies, or oversight mechanisms to guide antimicrobial stewardship across healthcare settings
Community-centered administrative strategies	Interventions designed to influence antibiotic use at the community level through public engagement, awareness campaigns, and primary care-oriented stewardship initiatives
Prescription rationalization and dispensing regulation	Interventions targeting prescriber behavior to optimize antibiotic use through guidelines, restrictions, review mechanisms, and decision-making support at the point of care. Interventions targeting the dispensing and supply chain interface, including regulatory enforcement, pharmacy-level controls, and market-related influences that affect antibiotic access and distribution
Institutional accountability and premiums	Interventions implemented within healthcare organizations that enforce stewardship through internal policies, audit systems, performance monitoring, and accountability structures
Technology-driven education and capacity building	Interventions using digital platforms, electronic medical records, clinical decision support systems, or data-driven tools to guide, monitor, or optimize antimicrobial use
Infection prevention measures	Interventions implemented within healthcare organizations to ensure infection prevention and control

Based on six structured categories (Figure 3; Table 3), varied AMS interventions were reported across the countries in the included studies, reflecting key differences in several factors, including, but not limited to, health system governance and service delivery infrastructure, health workforce strength and capacity building, social and cultural synergy, and government support for integration of WHO’s National Action Plans (NAPs) for implementing AMS interventions. For instance, the majority of interventions in HICs were health-system administrative governance such as the United Kingdom (van der Werf et al., 2018) (Nathwani et al., 2012), the United States (Okere et al., 2022; Persell et al., 2016), Canada (McCreary et al., 2021), and the Netherlands (Welschen et al., 2004; Wentzel et al., 2014); community-centered interventions such as in New Zealand (Chinzowu et al., 2023), Russia (Rachina et al., 2024), Australia (Wutzke et al., 2007), Canada (McKay et al., 2011), and the United States (Gonzales et al., 2005); institutional accountability and premiums, for example, the United Kingdom (Ashiru-Oredope et al., 2013; Bou-Antoun et al., 2018; Balinskaite et al., 2019), and the Netherlands (Welschen et al., 2004; van der Velden et al., 2016); and technology-driven capacity building interventions, such as Norway (Gjelstad et al., 2013), Poland (Wojkowska-Mach et al., 2018), Spain (Fernández Urr et al., 2014; Fernández-Urrusuno et al., 2020), the Netherlands (Welschen et al., 2004; de Greeff et al., 2016), and Canada (Weiss et al., 2011), attributed to strong and well-established healthcare infrastructure, well-placed governance system, and regulatory and institutional strength in HICs. Included studies showed that UMICs utilize hybrid action plans that amalgamate policy enforcement with capacity building to implement AMR interventions. For example, in Indonesia (Wiweko et al., 2016), the integration of digital health and mobile-based interventions was associated with improved antibiotic use practices and reduced misuse. In China, hospitals limited the procurement of antibiotics to 50 agents and lowered prescription targets (Ma et al., 2016), and they enforced a ban on IV antibiotics in outpatient dispensing (Wang et al., 2020a), which improved antibiotic use by 18% and decreased antibiotic prescription proportions, respectively. South Africa successfully improved AMS pharmacist-led hang-time (reducing time to first antibiotic dose), resulting in lower antibiotic use and higher de-escalation rates (Messin et al., 2015). Turkey, through the national antibiotic restriction program (NARP) campaign, increased awareness, capacity, and implementation of AMR and antimicrobial stewardship programs (ASPs) (Altunsoy et al., 2011).

**TABLE 3:** Summary of the included studies.

Reference	Country	Methodology			Identified administrative AMR interventions						Impact of interventions				
​	​	Qualitative	Quantitative	Mix	Health-system governance	Community-centered strategies	Prescription and dispensing regulation	Accountability and premiums	Education and capacity building	Infection prevention measures	Judicious consumption	Prescription rationalization	Dispensing regulation	Enhanced community awareness	Capacity building
Welschen et al. (2004)	Netherlands	​	X	​	X	​	​	​	​	​	X	X	​	​	​
Rattanaumpawan et al. (2010)	Thailand	​	X	​	X	​	​	​	​	​	​	X	​	​	​
Catry et al. (2018)	Belgium	X	​	​	X	​	​	​	​	​	​	X	​	​	X
Wiweko et al. (2016)	Indonesia	X	​	​	X	​	​	​	​	​	X	​	​	​	​
Ma et al. (2016)	China	​	X	​	X	​	​	​	​	​	X	X	​	​	​
Bremmer et al. (2018)	United States	X	​	​	X	​	​	​	​	​	X	​	​	​	​
Kim et al. (2016)	India	​	X	​	X	​	​	​	​	​	X	​	​	​	​
Messin et al. (2015)	South Africa	​	X	​	X	​	​	​	​	​	X	​	​	​	​
Okere et al. (2022)	United States	​	X	​	X	​	​	​	​	​	X	​	​	​	​
Gebretekle et al. (2020)	Ethiopia	​	X	​	X	​	​	​	​	​	​	X	​	​	​
Molina et al. (2019)	Spain	​	X	​	X	​	​	​	​	​	X	​	​	​	X
Cona et al. (2020)	Italy	​	X	​	X	​	​	​	​	​	X	​	​	​	​
Sacco et al. (2018)	United States	​	X	​	X	​	​	​	​	​	X	​	​	​	​
McCreary et al. (2021)	Canada	X	​	​	X	​	​	​	​	​	X	​	​	​	​
Park et al. (2005)	Korea	​	X	​	X	​	​	​	​	​	​	X	​	​	X
Wang et al. (2020a)	China	​	​	X	X	​	​	​	​	​	​	X	​	​	​
Wentzel et al. (2014)	Netherland	X	​	​	X	​	​	​	​	​	​	X	​	​	​
Persell et al. (2016)	United States	​	X	​	X	​	​	​	​	​	X	​	​	​	X
van der Werf et al. (2018)	England	​	X	​	X	​	​	​	​	​	​	X	​	​	​
Donà et al. (2018)	Italy	​	X	​	X	​	​	​	​	​	​	X	​	​	​
Kamalo et al. (2023)	Malawi	X	​	​	X	​	​	​	​	​	​	X	​	​	​
Altunsoy et al. (2011)	Turkey	​	X	​	X	​	​	​	​	​	X	​	​	X	X
Nathwani et al. (2012)	Scotland	​	X	​	X	​	​	​	​	​	X	​	​	​	​
Lee et al. (2014)	South Korea	​	X	​	X	​	​	​	​	​	​	X	​	​	​
Chinzowu et al. (2023)	New Zealand	​	X	​	​	X	​	​	​	​	X	​	​	X	X
Mudenda et al. (2023)	Zambia	​	X	​	​	X	​	​	​	​	X	​	​	X	​
Rondon et al. (2023)	Peru	​	X	​	​	X	​	​	​	​	​	X	​	X	​
Huong et al. (2021)	Vietnam	X	​	​	​	X	​	​	​	​	X	​	​	X	X
Talaat et al. (2014)	Egypt	​	X	​	​	X	​	​	​	​	X	​	​	X	​
Rachina et al. (2024)	Russia	X	​	​	​	X	​	​	​	​	X	​	​	X	​
Wutzke et al. (2007)	Australia	​	​	X	​	X	​	​	​	​	X	​	​	X	​
McKay et al. (2011)	Canada	X	​	​	​	X	​	​	​	​	​	X	​	X	​
Plachouras et al. (2014)	Greece	​	X	​	​	X	​	​	​	​	​	X	​	X	X
Gonzales et al. (2005)	United States	​	X	​	​	X	​	​	​	​	​	​	​	X	X
Formoso et al. (2013)	Italy	​	X	​	​	X	​	​	​	​	​	X	​	X	​
Sabuncu et al. (2009)	France	​	X	​	​	X	​	​	​	​	​	X	​	X	X
Lo et al. (2021)	Hong Kong	​	​	X	​	X	​	​	​	​	​	​	​	X	X
Fernandes et al. (2019)	India	​	X	​	​	X	​	​	​	​	​	​	​	X	​
Little et al. (2014)	United Kingdom	​	X	​	​	​	X	​	​	​	X	​	​	​	​
Wang et al. (2020b)	China	​	X	​	​	​	X	​	​	​	X	​	​	​	​
Galimam et al. (2022)	Australia	​	X	​	​	​	X	​	​	​	X	​	​	​	​
Gulliford et al. (2019)	United Kingdom	​	X	​	​	​	X	​	​	​	​	X	​	​	​
Mattos et al. (2017)	Brazil	​	X	​	​	​	X	​	​	​	​	​	X	​	​
Moura et al. (2015)	Brazil	​	X	​	​	​	X	​	​	​	​	​	X	​	​
Lopes-Júnior et al. (2015)	Brazil	​	X	​	​	​	X	​	​	​	​	​	X	​	​
Wirtz et al. (2013)	Chile	​	X	​	​	​	X	​	​	​	​	​	X	X	​
Marković-Peković et al. (2017)	Bosnia and Herzegovina	​	​	X	​	​	X	​	​	​	​	​	X	X	​
Abilova et al. (2018)	Azerbaijan	​	X	​	​	​	X	​	​	​	​	​	X	X	​
Ivanovska et al. (2018)	North Macedonia	​	X	​	​	​	X	​	​	​	​	​	X	X	​
Chalker et al. (2005)	Vietnam	​	​	X	​	​	X	​	​	​	​	​	X	​	X
Arparsrithongsagul et al. (2015)	Thailand	​	​	X	​	​	X	​	​	​	​	​	X	​	X
Hayes et al. (2022)	England	​	​	X	​	​	X	​	​	​	​	​	X	X	X
Farooqui et al. (2020)	India	​	X	​	​	​	X	​	​	​	​	​	X	​	​
Alrasheedy et al. (2020)	Saudi Arabia	​	​	X	​	​	X	​	​	​	​	​	X	​	​
Al-Jedai et al. (2022)	Saudi Arabia	​	X	​	​	​	X	​	​	​	​	​	X	​	​
van der Velden et al. (2016)	Netherlands	​	X	​	​	​	​	X	​	​	​	X	​	​	X
Ashiru-Oredope et al. (2013)	England	​	X	​	​	​	​	X	​	​	​	X	​	​	X
Johnston et al. (2019)	Northern Ireland	​	X	​	​	​	​	X	​	​	​	X	​	X	X
Xiao et al. (2013)	China	X	​	​	​	​	​	X	​	​	​	X	​	​	​
Roberts et al. (2015)	United Kingdom	​	X	​	​	​	​	X	​	​	​	X	​	​	​
Bou-Antoun et al. (2018)	England	​	X	​	​	​	​	X	​	​	​	X	​	​	​
Balinskaite et al. (2019)	England	​	X	​	​	​	​	X	​	​	​	X	​	​	​
Yip et al. (2014)	China	​	X	​	​	​	​	X	​	​	​	X	​	​	​
Høgli et al. (2016)	Norway	​	X	​	​	​	​	X	​	​	X	​	​	​	X
Thampi et al. (2019)	Canada	​	X	​	​	​	​	X	​	​	X	​	​	​	​
Gjelstad et al. (2013)	Norway	​	X	​	​	​	​	​	X	​	​	X	​	​	X
Wojkowska-Mach et al. (2018)	Poland	​	X	​	​	​	​	​	X	​	​	​	​	X	X
Weiss et al. (2011)	Canada	​	X	​	​	​	​	​	X	​	X	​	​	​	X
de Greeff et al. (2016)	Netherlands	X	​	​	​	​	​	​	X	​	X	​	​	​	​
Butler et al. (2012)	United Kingdom	​	X	​	​	​	​	​	X	​	X	​	​	​	​
Laks et al. (2019)	Brazil	​	X	​	​	​	​	​	X	​	X	​	​	​	X
Fernández Urr et al. (2014)	Spain	​	X	​	​	​	​	​	X	​	​	X	​	​	​
Fernández-Urrusuno et al. (2020)	Spain	​	X	​	​	​	​	​	X	​	X	​	​	​	​
Stuart et al. (2014)	Australia	​	​	X	​	​	​	​	​	X	X	​	​	​	​
Lean et al. (2019)	United Kingdom	​	X	​	​	​	​	​	​	X	X	​	​	​	​
Nkosi and Sibanda (2021)	South Africa	​	X	​	​	​	​	​	​	X	X	​	​	​	X

Although LMICs do not have well-established heath and governance systems compared to HICs, our included studies revealed that LMICs strategically utilized limited resources, cultural settings, and strong community networks to their advantage and compensated for relatively weaker enforcement capacity. For instance, in India (Kim et al., 2016), four secondary-care hospitals established an effective antimicrobial stewardship program using a hub-and-spoke model (a centralized system supporting peripheral facilities with stewardship expertise), which resulted in a marked reduction in infections and major antibiotic use. Through the incorporation of the National Institute for Health and Care Excellence (NICE) e-Bug module into school curricula in Malawi and India, AMR awareness was significantly improved (Fernandes et al., 2019). In Vietnam (Huong et al., 2021), market vendors and buyers were educated on antibiotic misuse, resulting in a 21% reduction in misuse. In Egypt, antibiotic use practices improved by 23% through mosque-based campaigns with monitoring (Talaat et al., 2014). Our review demonstrated that LICs utilized low-cost and scalable administrative antimicrobial stewardship interventions tailored to resource-limited settings to curb AMR. For example, implementation of drug use evaluation (DUE) with prior antibiotic authorization for high-risk antibiotics in Thailand reduced antibiotic prescribing for respiratory symptoms (Rattanaumpawan et al., 2010), Ethiopia’s pharmacist-led laboratory-supported intervention reduced antibiotic prescriptions (Gebretekle et al., 2020). Dispensing restrictions in Korea (Park et al., 2005) improved education and prescribing rationale. By engaging AMR officers, Malawi reduced broad-spectrum antibiotic prescriptions (Kamalo et al., 2023). South Korea implemented the antibiotic prescribing rate disclosure policy, which successfully reduced antibiotic prescriptions (Lee et al., 2014).

The findings of the review demonstrate that health-system administrative governance, community-centered administrative strategies, prescription rationalization and dispensing regulation measures, institutional accountability and premiums, technology-driven education and capacity building, and infection prevention measures are the most commonly implemented administrative antimicrobial stewardship interventions globally. Furthermore, most of these administrative interventions proved successful in producing positive outcomes in curbing AMR and judicious use of antimicrobials (Xiao et al., 2013; Stuart et al., 2014).

### 3.1 Health-system administrative governance

Health-system administrative governance initiatives are the most frequent interventions in the included studies, highlighting their pivotal role in AMS. A number of included studies demonstrated that administrative interventions encompassing leadership-led strategies, regulatory enforcement, and collaborative team-based approaches not only reduced inappropriate antibiotic consumption but also promoted their judicious use. Institutional leadership directives such as antibiotic selection consensus (Welschen et al., 2004), digital health-enabled stewardship interventions in Indonesia (Wiweko et al., 2016), introduction of integrative medicine in GP practice (van der Werf et al., 2018), and limitation of the procurements to 50 antibiotics (Ma et al., 2016) lowered the rate of antibiotic prescribing. Other institutional decisions that resulted in reduced rates of antibiotic use included daily, rather than weekly, infectious disease consultation (IDC) (Cona et al., 2020), ensuring penicillin skin testing (PST) (Sacco et al., 2018), and the implementation of a clinical pathway (CP) for community-acquired pneumonia (Donà et al., 2018). The success of health-system administrative governance in controlling AMR depends on fostering collaborative AMS teams to coordinate activities, and it is evident from multiple included studies, which showed that pharmacist-led pre-authorization, drug use evaluation (DUE) for high-risk antibiotics, IV antibiotic hang-time protocol, medication therapy management (MTM) services, and laboratory-supported interventions (Rattanaumpawan et al., 2010; Messin et al., 2015; Okere et al., 2022; ;Gebretekle et al., 2020) resulted in decreased antimicrobial use. Other collaborative efforts in the included studies revealed that quarterly general practitioner (GP-peer discussion groups (Catry et al., 2018), the hub-and-spoke model for implementing ASPs (Kim et al., 2016), merging IDC and an educational ASP team (Molina et al., 2019), in-person educational rounds (McCreary et al., 2021), participatory development through user evaluations (Wentzel et al., 2014), and the establishment and engagement of AMR officers and local antimicrobial teams (Nathwani et al., 2012) not only reduced antibiotic use and misuse but also enhanced antibiotic de-escalation and prescribing reasoning skills.

Enforcement of regulatory measures as a part of health-system administrative governance improved antibiotic prescription trends. As evident from one of the included studies (Bremmer et al., 2018), antimicrobial stewardship interventions integrated into electronic medical records, such as antibiotic restrictions and time-out mechanisms, are associated with improved prescribing practices and optimized antimicrobial use, which may indirectly contribute to improved clinical outcomes. Other antibiotic prescription regulations, such as accountability and justification on antibiotic intervention (Persell et al., 2016) and implementation of antibiotic prescribing rate disclosure policy (Lee et al., 2014), along with national antibiotic restriction program (NARP) (Altunsoy et al., 2011) and the ban on IV antibiotics in the outpatient department (Wang et al., 2020a), resulted in decreased antibiotic prescribing and overall usage.

### 3.2 Community-centered administrative strategies

Beyond institutional interventions, community engagement empowers the public with knowledge and shapes behaviors toward responsible antibiotic consumption. A wide range of community-based administrative stewardship initiatives, ranging from the national to the school level, were identified. These initiatives leveraged multifaceted strategies and platforms to promote awareness and judicious use of antibiotics in the community. A number of included studies showed that blended outdoor education on antibiotic use (Chinzowu et al., 2023), radio-based awareness campaigns targeting rural populations (Mudenda et al., 2023), and national campaigns against self-medication (Rondon et al., 2023) demonstrated measurable impacts, with improvements in antibiotic use. Some of the included studies showed that interventions targeted at specific societal groups proved impactful. Multiple included studies revealed that targeted antibiotic misuse education for market vendors and buyers (Huong et al., 2021), mosque-based antibiotic education campaigns (Talaat et al., 2014), and cultural poetry contests on antibiotic responsibility (Rachina et al., 2024) enhanced community awareness and engagement. A multifaceted campaign targeting parents of schoolchildren on the judicious use of antibiotics (Plachouras et al., 2014) promoted shifts toward narrow-spectrum antibiotic use, even when total consumption remained unchanged. Another included study revealed household- and office-based educational intervention (Gonzales et al., 2005). Incorporating NICE e-Bug module into secondary school students’ curriculum enhanced their understanding of AMR spread factors (Fernandes et al., 2019), while student-led AMR campaigns raised awareness among elderly patients (Lo et al., 2021).

### 3.3 Prescription regulation measures

Other administrative interventions identified during our review included prescription regulation. These regulations emphasized administrative implementation of the delayed prescribing model (Gjelstad et al., 2013), post-dated and collection-only script (Gjelstad et al., 2013), controlled prescription program (Gjelstad et al., 2013), restrictive-prescribing stewardship (Gjelstad et al., 2013), and inclusion of antibiotic hardstop (AH) in electronic prescribing (Galimam et al., 2022) (Wojkowska-Mach et al., 2018).

### 3.4 Institutional accountability and premiums

Regular institutional monitoring and audit systems based on antibiotic consumption and AMR trends play a crucial role in supporting healthcare systems in rationalizing antibiotic use. This includes pharmacist-led monitoring and feedback systems based on antibiotic consumption quality indicators (Wojkowska-Mach et al., 2018; Murray et al., 2022), the introduction of audit mechanisms of patient charts within accreditation programs (Naghavi et al., 2024; Baur et al., 2017), as outlined in one of the included studies (Basnyat et al., 2015), along with mandated targets, task forces, audits, and inspections by the Chinese ministry. Implementation of these systems not only improved antibiotic prescribing quality but also decreased antibiotic sales and reduced the percentage of prescriptions. In addition to these, the introduction of incentives and quality premiums (Bou-Antoun et al., 2018; Bou-Antoun et al., 2018), pay-for-performance strategies, and premium provisions (Bou-Antoun et al., 2018) reduced antibiotic prescriptions. As indicated by one of the included studies (Balinskaite et al., 2019), financial incentives for local healthcare commissioners improved quality and reduced antibiotic prescribing in primary care.

### 3.5 Technology-driven education and capacity building

Capacity building of healthcare professionals (HCPs) through targeted education and digital platform initiatives is one of the most common administrative interventions identified in the included studies. Health-system administrative decisions to incorporate targeted education modalities (Weiss et al., 2011), such as peer detailing (WHO, 2021), continuous medical education (Wojkowska-Mach et al., 2018), and digital platforms, including flowcharts, decision support (Weiss et al., 2011), health applications (Weiss et al., 2011), and web-based courses (Weiss et al., 2011), provide HCPs with valuable hands-on information about AMR and the judicious consumption of antibiotics. One such study (Gjelstad et al., 2013), a randomized controlled trial of the Stemming the Tide of Antibiotic Resistance (STAR) educational program, resulted in reduced antibiotics without impacting safety.

### 3.6 Infection prevention strategies

Strong infection prevention and control (IPC) initiatives can be one of the most effective ways to control the spread of AMR in a healthcare setting since every avoided infection reduces the need for antimicrobial intervention and supports the judicious use of antibiotics. IPC initiatives, such as enforcing cleaning and disinfection practices, implementing hand hygiene standard operating procedures (SOPs), and utilizing personal protective equipment, were the least frequently reported strategies in the included studies. One of the included studies (Lean et al., 2019) revealed that structured daily hydration rounds, involving scheduled staff-led fluid intake and staff training, were associated with a reduced incidence of urinary tract infections requiring antibiotics. Improved hydration may reduce infection risk, thereby indirectly decreasing the need for antibiotic use. Another study conducted in Australia (Lean et al., 2019) describes how the induction of an infection control nurse consultant for aged care stabilized infection rates, thereby reducing antibiotic use. Daily ward monitoring of antibiotic use, guided by hospital antibiotic guidelines and protocols and conducted by an IPC specialist, improved ASP implementation among intensive care unit (ICU) patients in a large academic hospital in South Africa (Moura et al., 2015).

## 4 Discussion

Over the past few years, the policy paradigm has shifted toward more enabling forms of governance and leadership, allowing broader professional decision-making autonomy while strengthening system-level administrative stewardship mechanisms (Adler and Borys, 1996; Glenngård and Ellegård, 2018). This should be interpreted through an administrative lens, as many interventions—while clinical or educational in their immediate function—derive their impact from the governance structures, policy mandates, and institutional leadership that enable their implementation and sustainability. The findings of this review suggest that the effectiveness of administrative AMS interventions is closely linked to the broader health-system context, including governance strength, resource availability, and community engagement. Although HICs tend to leverage institutional infrastructure and policy enforcement, LMICs achieve measurable impact through culturally tailored, low-cost, and community-centered strategies, highlighting the importance of context-sensitive implementation. This synthesis underscores that administrative stewardship is not merely supportive but acts as a critical enabler of clinical AMS, shaping outcomes by aligning policies, accountability, and education with systemic capacity and societal behaviors.

Health-system governance and leadership is crucial for successful implementation of AMS interventions. It provides an enabling environment for ASP by setting targets, enforcing legal mandates, and facilitating multisector coordination. In LMICs, issues such as infirm and incapacitated health systems, poorly regulated antibiotic dispensing, and financial constraints congregate to aggravate the AMR exigency (Dyar et al., 2017; Ehsan et al., 2023; Ayukekbong et al., 2017; Piddock, 2016; Gelband and Laxminarayan, 2015; O'Neill, 2016). Coherently, in Pakistan, the failure in combating AMR transcends the boundaries of clinical practice. It is rooted in governance failure and fundamentally reflects a dearth of commitment and lack of responsibility from government regulatory bodies, due to which policy discussions on the NAP have not translated into real-time implementation at regional and local levels; Saleem (2025) validated this point. Several studies demonstrate the necessity of impactful leadership to establish and enforce an effective quality improvement program (Scully, 2015; McSherry et al., 2012; Kruk et al., 2018; Stoumpos et al., 2023; Sfantou et al., 2017).

Leadership commitment is the primary element among the Centers for Disease Control and Prevention (CDC) seven core elements for the implementation of an ASP in a health-system setting (Control CfD and Prevention, 2014). Although several interventions identified in this review involve clinical, educational, or community-based activities at the operational level, their implementation, coordination, and sustainability were largely dependent on administrative leadership, governance structures, and institutional decision-making. Therefore, these strategies were categorized as administrative antimicrobial stewardship interventions because they are initiated, enabled, regulated, or scaled through policy-level or managerial actions within healthcare systems. Evidence shows that collaborative and governance-backed system-level interventions have resulted in a decline in community systemic antibiotic dispensing in New Zealand (Fanning et al., 2025). The same is indicated in our review; for instance, the administrative decision by hospital leadership to conduct quarterly GP peer discussion groups on sinusitis guidelines in Belgium improved outcomes and shortened treatment duration (Catry et al., 2018). Similarly, United States’ health-system governance decisions such as antibiotic restriction and time-out in electronic medical records (EMRs) (Bremmer et al., 2018), reduced antibiotic consumption, and pharmacist-led ASP with MTM service (Okere et al., 2022) resulted in 37.2% better IV hang-time compliance; enforcement of PST (Sacco et al., 2018) supported antimicrobial stewardship by enabling the use of appropriate first-line and narrower-spectrum antibiotics in patients with inaccurately reported penicillin allergies, and implanting accountable justifications for prescribing antibiotics (Persell et al., 2016) aligned needs, streamlined tasks, and fostered ownership and safety. In Spain, the hospital’s administration strategy, which combined infectious disease consultation and educational ASP interventions, lowered total antimicrobial use (Molina et al., 2019). In Italy, daily, rather than weekly, infectious disease (ID) consultation strategy reduced antibiotic consumption (Cona et al., 2020). The policy of inducting integrative or alternative medicine GPs in England is under consideration (van der Werf et al., 2018). This finding of our review is consistent with that of Aljarallah (2017), who demonstrated that herbal extracts and essential oils—which act through multiple inhibitory pathways against *Clostridium difficile* infection and make it harder for pathogens to develop resistance—represent a promising future alternative. In China, hospital leadership decided to limit the procurement of antibiotics to 50 agents, along with lowering prescription targets (Ma et al., 2016), and enforced a ban on IV antibiotics in the outpatient department (Wang et al., 2020a), which improved antibiotic use by 18% and decreased the proportion of antibiotic prescriptions, respectively. Turkey, through the NARP campaign, increased awareness, capacity, and implementation of AMR and ASP (Altunsoy et al., 2011). In India, a policy decision was taken to use a hub-and-spoke model to establish an effective antimicrobial stewardship program at four secondary-care hospitals, which resulted in marked reduction in infections and major antibiotic use (Kim et al., 2016).

As a critical enabler of systematic change through wider acceptance, administratively driven community engagement is increasingly acknowledged as a fundamental component of impactful public health interventions. Evidence from infectious disease control shows that community mobilization has significantly improved vaccination rates, hygiene practices, and adherence to treatment protocols (Gilmore et al., 2020). Another piece of evidence shows that civil society organizations in Africa have supported AMR prevention and control (Fraser et al., 2021). Another study reveals that community-based interventions (CBIs) are effective in reducing the prevalence of hookworm, *Trichuris trichiura*, and *Ascaris lumbricoides* (Ugwu et al., 2024). Antibiotic misuse is multisectoral, and misuse in the community is one of the main drivers of AMR. At the community level, according to Kardas et al. (2005), approximately one-fourth of patients stored leftover antibiotics for future use, while over one-third of patients did not adhere to their prescribed antibiotic regimen. This reflects inappropriate antibiotic use behavior (Kardas et al., 2005). One study (Li et al., 2016) demonstrates that a number of developing countries are facing the problem of over-the-counter sale of antibiotics without a prescription or medical advice. Another dimension of this issue is the continued utilization of antibiotics in the absence of awareness among the general public (Economou and Gousia, 2015). Since the increasing rate of resistance among community-acquired infections is not matched by the development of newer antibiotics (Bhagwati, 2010), there is an urgent need for community-centered awareness and behavioral reforms enforced through a responsive regulatory system and effective leadership strategies to curb AMR. The significance of community synergy is also evident from Rehman et al. (2017), who showed enhanced immunization coverage through community education and awareness regarding the importance of immunization in children.

Coherently, a significant discovery from our review identified a number of administratively enabled, community-centered, multistakeholder strategies that proved highly impactful in increasing societal awareness and promoting community behavioral modifications toward antibiotic utilization. For instance, a blended outdoor health education on antibiotic use in New Zealand improved antibiotic use by 17% (Chinzowu et al., 2023). A poetry contest on antibiotic responsibility in Russia increased awareness and engagement (Rachina et al., 2024). The “Do Bugs Need Drugs” program in Canada improved prescribing knowledge and antibiotic prescribing while stabilizing costs (McKay et al., 2011). In Greece, parent-directed educational interventions helped shape patient expectations and healthcare-seeking behavior, which indirectly supported more rational antibiotic prescribing practices; however, overall consumption remained unchanged (Plachouras et al., 2014). Household- and physician’s office-based education in United States proved impactful in improving patient education; however, the impact of physician intervention remained minimal (Gonzales et al., 2005). Local multifaceted campaigns in Italy reduced antibiotic prescribing (Formoso et al., 2013). Public education campaigns in France decreased antibiotic prescriptions by 26.5% (Sabuncu et al., 2009). National anti-self-medication outreach (community awareness to reduce non-prescription antibiotic use) in Peru resulted in a 22% decrease in unnecessary prescriptions (Rondon et al., 2023). Education of market vendors and buyers on antibiotic misuse in Vietnam reduced antibiotic misuse by 21% (Huong et al., 2021). Mosque-based campaigns with monitoring in Egypt improved antibiotic use practices by 23% (Talaat et al., 2014). The NICE e-Bug module in school syllabus in India improved AMR awareness (Fernandes et al., 2019). Radio campaigns for rural areas in Zambia increased awareness by 19% (Mudenda et al., 2023). Student-led AMR education for elderly patients in Hong Kong improved knowledge regarding antibiotics and AMR (Lo et al., 2021). Our findings are coherent with those of Birgand et al. (2018), who stated that a multi-stakeholder approach is one of the best possible methods for dealing with this problem. Drug utilization or prescription review is a crucial feature of healthcare service delivery that works to ensure rational prescribing and medication use across the entire patient cohort (Fan et al., 2023).

Establishing and enforcing standard operating procedures provides a structured framework for physicians, pharmacists, and healthcare administrators to obtain a strong evidence-base that supports rational prescription practices, ensuring drug safety, efficacy, rationality, and cost-effectiveness (Postema, 2020; Xie et al., 2022). Evidence shows a significant reduction in opioid prescribing under the influence of prescription restrictions (Cosler et al., 2025). Prescription regulation is the need of the hour to curb AMR. Currently, initiatives to strengthen prescribing-related antimicrobial stewardship mainly concentrate on identifying suitable “AMS champions” to motivate clinicians to prescribe responsibly and promote judicious antimicrobial use (Broom et al., 2021). However, prescription regulation measures, techniques, and enforcement by institutional leadership may be more impactful in addressing the challenges of AMS. Our review findings indicate that the delayed prescribing strategy in Norway reduced antibiotic prescribing for respiratory tract infections (RTIs) and broad-spectrum antibiotic use (Gjelstad et al., 2013). Delayed and conditional prescription strategies in the United Kingdom reduced antibiotic use (Little et al., 2014). Electronic support for antibiotic prescribing in the United Kingdom reduced antibiotic prescribing for RTIs (Gulliford et al., 2019). AH in e-prescribing in Australia resulted in shorter days of therapy (DOT) and length of therapy (LOT) (Galimam et al., 2022). Restrictive prescription stewardship in China reduced total antibiotic consumption by 32.6% (Wang et al., 2020b). The controlled prescription program of Zambia resulted in an appropriate use of antibiotics by 40.9% (Mudenda et al., 2023). Our review findings are coherent with those of De Vries et al. (2022), who stated that there was an increase in adherence to antibiotic prescribing guidelines from 19% to 47% and a resultant decreased consumption of amoxicillin, azithromycin, and flucloxacillin, as well as with those of Mustafa et al. (2020), who revealed improved prescribing from 41% to 73% by adhering to guidelines. Our findings are also consistent with those of von Pressentin et al. (2019), Brink et al. (2017), and Wasserman et al. (2017).

Monitoring, audit, and feedback in healthcare systems, intended to optimize provider service delivery while reducing costs and institutional accountability, have already been utilized to facilitate external accountability (Davies and Harrison, 2003; Funck, 2015). As identified in one study, the audit and feedback cycle not only resulted in visible improvements in primary healthcare physicians’ practices but also improved clinical skills and compliance with clinical protocols (Omair et al., 2025). Consistently, institutional accountability was identified as an impactful category in our review, where leadership-enforced accountability measures strengthened AMR interventions. For example, the monitoring and feedback system of the Netherlands (Welschen et al., 2004) decreased the rate of antibiotic prescribing; patient chart audits pre- and post-educational interventions in Northern Ireland (Johnston et al., 2019) improved antibiotic prescription quality; and the Chinese ministry’s mandated targets, taskforces, audits, and inspections lowered the sale and prescription percentage (Xiao et al., 2013). Our findings are consistent with those of Vanstone et al. (2022), who demonstrated that audit and feedback as monitoring interventions improved antibiotic prescribing by primary healthcare prescribers. Glenngård and Anell (2021) also noted that audits and feedback improve the quality of healthcare delivery. However, one of the included studies (Wojkowska-Mach et al., 2018) reported that in Poland, despite the use of quality indicator monitoring systems, rational antibiotic use remained suboptimal.

Apart from accountability, the provision of premiums and physicians’ pay-for-performance schemes have become prominent in many countries (Roland and Campbell, 2014; Song et al., 2019). This is coherent with our review findings; for example, in England, the introduction of a quality premium for GPs (Bou-Antoun et al., 2018) and financial incentives for local healthcare commissioners (Balinskaite et al., 2019) decreased prescriptions for RTIs and reduced antibiotic prescribing, respectively. Similarly, capitation with pay-for-performance in China resulted in a 15% reduction in antibiotic prescriptions (Yip et al., 2014). These findings are consistent with those of Islam et al. (2018), who reported that a reduction in antibiotic use was observed in acute hospitals across England following the introduction of a financially linked antibiotic prescribing quality improvement initiative, Commissioning for Quality and Innovation (AMR-CQUIN), which was associated with a reduction in antibiotic use.

Given the dynamic nature of modern healthcare, staying updated on new treatments and best practices is crucial for meeting patient needs (Fourré et al., 2022). Continuous education and training, both conventional and technology-assisted, are imperative for capacity building, which, in turn, is pivotal for healthcare providers and so is for patients (Miller et al., 2020; Afari-Asiedu et al., 2021). According to the WHO, outpatients with good information on AMR are likely to avoid the abuse and misuse of antibiotics (Shallcross and Davies, 2014). This review demonstrated that conventional and technology-driven education, training, and capacity building positively impacted ASP activities. For instance, peer academic detailing in Norway reduced prescribing for RTIs and broad-spectrum antibiotic use (Gjelstad et al., 2013). Education for GPs, pharmacists, and patients in the Netherlands reduced prescribing for respiratory symptoms (Welschen et al., 2004). Despite post-graduation and continuous education for physicians and pharmacists in Poland, rational antibiotic use remains suboptimal (Wojkowska-Mach et al., 2018). Education for physicians and pharmacists in Canada lowered outpatient antibiotic prescriptions (Weiss et al., 2011). Nurse-focused AMS tools in the Netherlands improved AMS through nurse tools (de Greeff et al., 2016). The STAR program of the United Kingdom reduced antibiotic use without impacting safety (Butler et al., 2012). Integration of training and digital guides into healthcare services in Spain increased appropriate antibiotic prescribing from 36% to 57% (Fernández Urr et al., 2014). mHealth apps in Indonesia improved antibiotic use by 18% (Wiweko et al., 2016). Web-based AMR courses for medical students in Brazil enhanced antimicrobial stewardship in medical education (Laks et al., 2019). Our findings are concordant with those of Miller et al. (2020) and Afari-Asiedu et al. (2021), who reported that education and awareness are pivotal for healthcare professionals and patients.

Healthcare-associated infections (HAIs) are the most common adverse event faced in any healthcare setting (Allegranzi et al., 2011). According to the WHO, in high- and middle/low-income countries, 3.5%–12% of hospitalized patients develop at least one HAI (Allegranzi et al., 2011). Particularly in LMICs, challenges such as sub-optimal infection prevention practices, restricted availability of personal protective equipment (PPE), incapacitated and overcrowded healthcare settings, and poor sterilization protocols accelerate the emergence of resistant pathogens (Huemer et al., 2020; Ghosh et al., 2020). According to Harbarth et al. (2003) and Umscheid et al. (2011), approximately 50% of these HAIs can be prevented. Infection prevention and control policies in hospitals are designed to reduce these predisposing factors and, hence, the burden of HAIs and, consequently, antimicrobial resistance. Institutional leadership’s commitment is essential to enforce and facilitate IPC activities. Our review findings reveal that administration-backed and leadership-led AMS and IPC activities can control infection rates, which, in turn, reduce the requirement for antibiotics and, in this way, successfully curb AMR. For example, a leadership decision of an old-age home in the UK involved the implementation of structured daily hydration rounds, involving scheduled staff-led fluid intake and staff training, which were associated with a reduced incidence of urinary tract infections requiring antibiotics. Improved hydration may reduce infection risk, thereby indirectly decreasing the need for antibiotic use (Lean et al., 2019). Similarly, Australia’s decision to involve infection control nurse consultants in aged care stabilized infection rates and reduced antibiotic use (Stuart et al., 2014). In South Africa, daily ward monitoring by an IPC specialist improved the impact of the ASP (Nkosi and Sibanda, 2021). These findings are consistent with those of Saint et al. (2010), who stated that hospital management commitment and impactful clinical leadership are necessary for successful implementation of IPC/AMS activities.

Although our review provides a novel and comprehensive mapping, the evidence landscape remains fragmented, requiring cautious interpretation. The lack of a standardized definition for administrative antimicrobial stewardship may have introduced variability in the classification of interventions. Heterogeneity of the included studies makes direct comparison of outcomes challenging. Although the study spans all WHO regions, there is limited representation of certain WHO regions, particularly the EMRO region, potentially limiting generalizability. Most studies originated from high-income countries, with fewer contributions from low- and middle-income countries, where stewardship challenges may be more acute. Google Scholar is less reproducible and can retrieve non-peer-reviewed material. Additional databases such as EMBASE (Excerpta Medica dataBASE), Web of Science, Scopus, and CINAHL (Cumulative Index of Nursing and Allied Health) would have strengthened comprehensiveness. The review may over-represent successful interventions. Administrative and governance-related interventions are deeply context-specific, which may limit the applicability of findings across diverse health systems.

Interestingly, this review identified a number of gaps in administrative AMR, which may hinder the achievement of optimal AMR control. These include weak political ownership with unclear responsibilities of AMS at the government level, absence of standardized administrative indicators and monitoring frameworks, limited financing and a lack of sustainable resource allocation, weak regulatory enforcement of antibiotic prescribing and dispensing, minimal integration with IPC, insufficient community and civil society engagement, and over-reliance on short-term donor-driven projects in LMICs.

However, these gaps can be addressed by elevating AMS as a national health security priority with designated responsibilities and by developing measurable performance indicators (e.g., governance scorecards and accountability metrics) to assess the performance of administrative AMS interventions. It can be achieved by creating dedicated AMS budget lines and integrating stewardship into health-system financing, strengthened by regulatory frameworks, e.g., enforcing prescription-only antibiotic sales. An association between hospital accreditation and licensing with ASPs and IPC implementation can ensure a holistic reduction in infection burden and antimicrobial demand. Fostering public–policy partnerships through awareness campaigns, patient advocacy, and involvement of civil society organizations in stewardship oversight can build locally owned, sustainable stewardship models.

## 5 Conclusion

This scoping review maps administrative and leadership-level AMS interventions globally. Beyond the clinical and individualistic approach, our scoping review explicitly focuses on administrative and leadership dimension of AMS interventions, an area that has remained fragmented and underexplored. This review has certain limitations, including reliance on only two databases and the predominance of studies from high-income countries, which may affect generalizability. To mitigate selection bias and enhance methodological rigor, predefined inclusion and exclusion criteria were applied, and the review followed PRISMA-ScR guidelines. Despite these limitations, the review identifies actionable administrative strategies to strengthen AMS, underscores the importance of governance, leadership, and community engagement, and provides a conceptual framework to inform future implementation and policy development. By clustering interventions into six thematic categories, this article provides a conceptual framework that connects otherwise siloed evidence into a coherent synthesis.

Overall, these interventions were associated with reductions in the antibiotic misuse, judicious prescribing, improvements in adherence to guidelines, and robust stewardship infrastructure. The results of this study can serve as a valuable resource for policymakers by highlighting that administrative antimicrobial stewardship is the cornerstone of impactful AMS, empowering clinical practices to translate into system-wide and sustainable change. It provides a way forward for the advancement of the stewardship discourse beyond “what clinicians must do” to “what systems must enable”—a necessary shift for meaningful progress against antimicrobial resistance. Notably, governance interventions, prescription rationalization, and community-centered strategies were most frequently associated with improvements in stewardship outcomes. Collectively, these findings highlight administrative antimicrobial stewardship as an area that remains fragmented and underexplored but holds substantial potential for strengthening global antimicrobial stewardship efforts.
